# The nexus between forest fragmentation in Africa and Ebola virus disease outbreaks

**DOI:** 10.1038/srep41613

**Published:** 2017-02-14

**Authors:** Maria Cristina Rulli, Monia Santini, David T. S. Hayman, Paolo D’Odorico

**Affiliations:** 1 Department of Civil and Environmental Engineering, Politecnico di Milano, Milan, Italy; 2 Division on Impacts on Agriculture, Forests and Ecosystem Services, Fondazione Centro Euro-Mediterraneo sui Cambiamenti Climatici, Viterbo, Italy; 3 Molecular Epidemiology and Public Health Laboratory (mEpiLab), Hopkirk Research Institute, Massey University, Private Bag 11 222, Palmerston North 4442, New Zealand; 4 Department of Environmental Sciences, University of Virginia, Charlottesville, VA, USA; 5 Department of Environmental Science, Policy and Management, University of California, Berkeley, CA, USA; 6 National Social Environmental Synthesis Center, University of Maryland, Annapolis, MD, USA

## Abstract

Tropical forests are undergoing land use change in many regions of the world, including the African continent. Human populations living close to forest margins fragmented and disturbed by deforestation may be particularly exposed to zoonotic infections because of the higher likelihood for humans to be in contact with disease reservoirs. Quantitative analysis of the nexus between deforestation and the emergence of Ebola virus disease (EVD), however, is still missing. Here we use land cover change data in conjunction with EVD outbreak records to investigate the association between recent (2004–2014) outbreaks in West and Central Africa, and patterns of land use change in the region. We show how in these EVD outbreaks the index cases in humans (i.e. spillover from wildlife reservoirs) occurred mostly in hotspots of forest fragmentation.

It has been argued that the 2013–2015 Ebola virus disease (EVD) outbreak in West Africa began due to deforestation^1^,^2^, yet environmental factors controlling the transmission (aka ‘spillover’) of ebolaviruses from wildlife reservoirs to humans are not well understood^2^,^3^,^4^,^5^,^6^,^7^. Some frugivorous and insectivorous bat species are suspected to serve as reservoirs for filoviruses, including viruses from the genus *Ebolavirus* such as Ebola virus (*Zaire ebolavirus*)^8^,^9^,^10^,^11^. It is believed that transmission to humans occurs either directly through contact with these reservoirs or indirectly through larger wildlife prone to ebolavirus infection (e.g., duikers and apes)^8^,^10^,^12^. Human encroachment in forested areas leads to forest destruction, habitat fragmentation, and may increase exposure to other zoonotic infections^13^ (e.g., Nipah virus, retroviruses^14^,^15^) through interactions with wildlife reservoir species resulting from direct contact (e.g. bushmeat consumption) as well as potentially decreasing biodiversity^16^,^17^. Human encroachment into forested areas may also enhance exposure to vector-borne diseases, including as leishmaniasis^18^, malaria^19^,^20^,^21^,^22^,^23^, and dengue^24^. This is a relatively novel aspect in the study of ecology of zoonotic infections that has proposed, but rarely quantified (but see ref. 15), while the case of other viruses still needs to be investigated. Our study shows that in the case of African ebolaviruses, spillover is more likely to occur in areas affected by forest fragmentation.

In the case of the 2013–2015 Ebola virus epidemic in West Africa there is no evidence of an infection in larger wildlife and it is also unclear whether the spillover from reservoir species to humans was caused by contact or bushmeat consumption^11^. It is reported that the index case for the 2013 Ebola virus epidemic in West Africa was a boy from Meliandou^11^,^25^, a village close to Gueckedou (Guinea). This village is not in the proximity to core forest areas but surrounded by a landscape strongly reshaped by plantations and other human activities. The geographic distribution of potential bat hosts is consistent with the distributions of ebolaviruses in Africa and antibodies against ebolaviruses suggestive of natural *Ebolavirus* infection have been found within those bat populations^8^,^12^,^26^,^27^. Along with related filoviruses from the *Marburgvirus* genus being isolated from fruit bats^28^, Ebola virus RNA has been isolated from bats^10^. Thus, bats are considered the putative reservoir hosts for ebolaviruses. The zoonotic niche of the EVD was recently mapped^8^, providing a low-resolution identification of areas at higher risk of *Ebolavirus* transmission to humans. Other studies have related spillover events to population density and vegetation cover^29^. Those efforts, however, did not account for the role of fine-scale landscape heterogeneity, forest fragmentation, and human encroachment in forested areas as a result of deforestation. Here we provide a quantitative assessment of the possible nexus existing between EVD outbreaks and forest loss and fragmentation in West and Central Africa^1^,^13^, two regions recently affected both by EVD outbreaks and the clearing and fragmentation of forested land (Figs 1, 2).

## Results and Discussion

**Figure 1:**
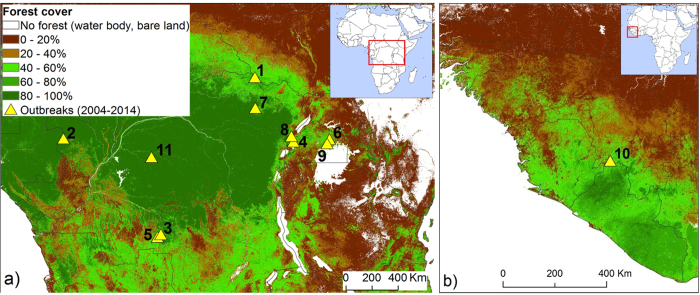
Forest cover maps and locations of first infection events in humans. Forest cover maps and locations of independent first infection events in humans (triangles, see Table 1) in Central (**a**) and West (**b**) Africa. The insets indicate the two African regions considered in this study. Legend in (**b**) is the same than in (**a**). Maps generated by the authors using ARCGIS 10.2-Version 10.2.0.338, licensed to Politecnico di Milano. The license term can be found on the following link: http://www.esri.com/legal/software-license.

High-resolution forest data from satellites are available since the year 2000^30^. To allow evaluation of changes in forest cover and forest fragmentation prior to each outbreak, we consider events of first reported *Ebolavirus* infections in humans (index cases) that occurred after 2004. We identify eleven independent index cases (Table 1)^8^,^31^: i.e., presumed primary infection events due to spillover from wildlife reservoirs to humans in the study region (triangles in Fig. 1). Using existing 30 m high-resolution tree cover data^30^, we find that on the outbreak year (Table 1) the average forest cover in the surroundings of these eleven centers of first infection (e.g., within a 25 or 50 km radius) was significantly greater than the average forest cover across the region (p-value 0.0052 and 0.0301; see Tables 2 and S1). While the centers of first infection are not preferentially located in hotspots of forest loss (Tables 2 and S2), they tend to occur in areas where on the outbreak year the average degree of forest fragmentation (e.g., within a 25 km, 50 km or 100 km distance from the infection center) was significantly higher (p-values 0.0062, 0.0047 and 0.0072, respectively) than in the rest of the region (Table S3). We analyzed forest fragmentation, in this study expressed in terms of a compound fragmentation index, CFI, defined as the fraction of the landscape covered with forest margin sites or with smaller (<200 ha) forest fragments (see Methods). Forest fragmentation (i.e., CFI) on average increases with decreasing distance from the center of infection (Figure S1 and Table S3). Likewise, the increase in forest fragmentation (between 2000 and the infection year) was on average stronger in areas close to the infection centers (Table S4). Within 25 km from the centers of first infection changes in forest fragmentation between 2000 and the infection year were on average positive (Table S4) and significantly greater than the average increase (2000–2014) in forest fragmentation across the region. These p-value estimates are conservative because, while in the infection areas changes in forest cover and fragmentation were evaluated between 2000 (baseline year) and time of infection, regional changes were determined using a longer period (2000–2014) during which fragmentation has increased across the region (Tables 2 and S2, S4). These results, however, could be affected by a bias because areas that are more populated are more likely to exhibit both enhanced contact between infection reservoir and humans and forest fragmentation by land use change^29^. Using spatially extended population data (see Methods), we find that population density within 25 km from the (presumed) first infection points is indeed significantly greater than across the region (p-value 0.0117) (Table 2). To remove the bias associated with higher population density, we compare forest cover, loss, and fragmentation between the areas surrounding the 11 centers of first infection (e.g., 25 km radius), and randomly selected areas (hereafter called IQR areas) with 25 km radius and population density comprised within the interquartile range (IQR) of the population in the areas of first infection. Interestingly, we found that while the mean population density within 25 km from the 11 centers of first infection and across the randomly selected IQR areas were not significantly different (p-value 0.5766), a significant difference existed in forest cover (p-value 0.0001), forest fragmentation (p-value 0.0318) and change in fragmentation (p-value 0.0033) between 2000 and the infection year. Similar level of significance are obtained when only sites of first infection of Central Africa are considered. Thus, sites of first infection on average exhibit significantly higher population density (Table 2), average forest cover (Tables 2 and S1), fragmentation and increase in fragmentation (Tables 2 and S3, S4) than the rest of the region. These findings are robust with respect to possible biases associated with non-uniform population densities. Interestingly, outbreaks occurred more often in forested areas affected by fragmentation, when considering areas with similar population density. Thus, even though the rates of forest loss in the areas of first infection are not significantly greater than those observed across the region as a whole, our results indicate that *Ebolavirus* spillover events from wildlife reservoirs to humans preferentially occur in areas that are relatively populated and forested, yet where deforestation is reshaping the forest boundaries by increasing forest fragmentation^29^. We recognize that since humans may travel long distances, the site of the first reported (index) case of EVD in an outbreak does not necessarily coincide with the site of first infection. For this reason, a neighborhood within a distance of at least 25 km was considered for each presumed center of first infection.

**Figure 2:**
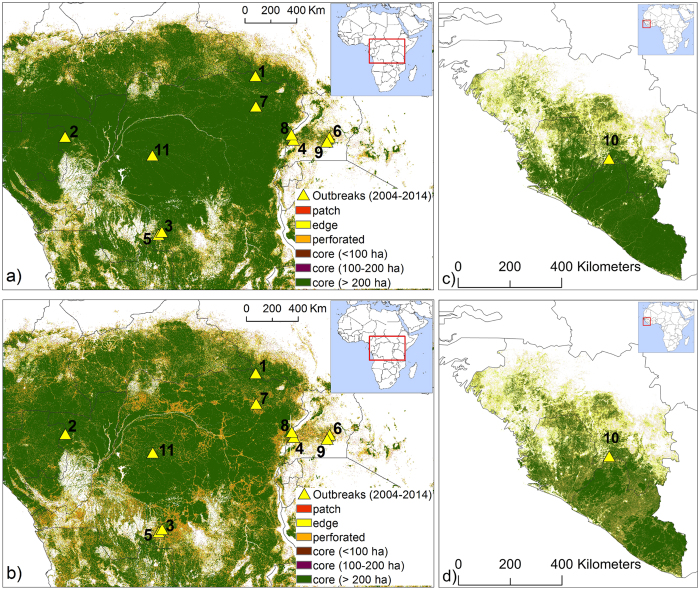
Forest fragmentation in Central and West Africa. Forest fragmentation in Central (panels a, and b) and West Africa (Panels c and d) in 2000 (top panels) and 2014 (bottom panels). Fragmentation is measured by the number of patch, edge, perforated and smaller forest areas (<200 ha). Most of the centers of first infection (yellow triangular markers) are located in areas affected by increasing forest fragmentation (see Table S3, S4). Maps generated by the authors using ARCGIS 10.2-Version 10.2.0.338, licensed to Politecnico di Milano. The license term can be found on the following link: http://www.esri.com/legal/software-license.

We also use the Getis-Ord analysis (see Methods) to determine whether the centers of first infection are hotspots of forest fragmentation and find (Table S3) that 8 out of the 11 infection events included in this study took place in fragmentation hotspots identified with confidence levels ranging between 90% and 99%. The three exceptions are the outbreaks of Yambio (Fig. 2 – event n. 1), which falls, however, very close (≈80 km) to a high fragmentation NW-SE corridor (Figure S2), and Odzala and Inkanamongo (Fig. 2 – events n. 2 and 11), the former associated with hunting/poaching activities in the forest and for which the source species remains uncertain^32^. Interestingly, both index cases 2 and 11 were reportedly thought to be infected while hunting small animals for food.

Overall our results are consistent with the notion that the transmission of ebolaviruses to human populations is more likely to occur in highly disturbed forested areas. Though it is unlikely that deforestation overall improves the habitat of bat species, ‘edge effects’ as a result of habitat fragmentation have been linked to a reduction in insectivorous and increase in frugivorous bat abundance in numerous studies^33^,^34^,^35^,^36^. In a recent systematic review of responses of tropical bats to habitat fragmentation, logging, and deforestation^37^ only two studies out of 117 were from Africa, precluding any analysis. One generality from this meta-analysis, however, was that frugivorous tropical bats often increase in fragmented habitats, though the studies were typically from the Neotropics^38^,^39^,^40^. In the absence of virus isolation from bats there is no conclusive evidence that bat species are the natural reservoirs for ebolaviruses and factors controlling the mechanisms of spillover to humans remain poorly understood^8^,^12^. However, our results are robust to any specific assumption on reservoir hosts, provided that the reservoir host is a forest dwelling wild species. While the reservoir hosts for ebolaviruses are still uncertain (see ref. 10,12), several index human cases of EVD, particularly in Gabon, had been linked to contact with Ebola virus infected apes (e.g. Gabon, 1996, 2001–2003, refs 41, 42, 43). Interestingly 64 animal carcasses were within a 2-hour walking distance of villages, including 22 gorillas (13 positive), eight chimpanzees (four positive), and six duikers (one positive)^42^. Furthermore, the impact of fragmentation and habitat use on these species is better studied than on bats. For example, following disturbance through logging in northern Republic of Congo, gorilla, chimpanzee and duiker densities initially decline but can all increase in density with time, sometimes exceeding pre-disturbance densities depending on the species^44^. Duiker more quickly increase in abundance peaking around 10 years post disturbance then decline, whereas chimpanzees and gorillas have been recorded to steadily increase with time over 30 years periods^44^. Together these studies suggest that habitat fragmentation facilitate EVD outbreaks as it may lead to increased contact between humans as they encroach and potent *Ebolavirus* reservoirs. Thus, fragmented forest edges could be preferential corridors for pathogen transmission from wildlife reservoirs to humans and thereby favor the emergence of some zoonotic infections^7^.

High degrees of forest fragmentation and their increase over time can be good indicators of enhanced opportunities for human contact with wildlife because of human penetration in wildlife habitat and, possibly, also improved habitat for some reservoir species^5^,^13^,^33^,^34^,^35^,^36^,^45^. In fact, while wildlife virus hosts vary in their sensitivity to habitat disturbance, the specific African bat species with the strongest evidence for being filovirus hosts (*Rousettus aegyptiacus* [for *Marburgvirus*], and *Hypsignathus monstrosus, Epomops franqueti*, and *Myonycteris torquata* [for *Ebolavirus*]) appear to exhibit a broad habitat tolerance^46^. Indeed, two of the putative Ebola virus hosts (i.e., *M. torquata* and *E. franqueti*) are often associated with primary and secondary forests as well as forest-grassland mosaic habitats^46^. Likewise, as noted above, gorilla, chimpanzee and duiker have been observed to increase in abundance after forest disturbance. Thus, it could be argued that while disturbance by deforestation destroys the habitat of specialist species, generalists – possibly including reservoirs of some zoonotic pathogens – thrive^4^,^5^,^6^, thereby further enhancing the risk of infection in human populations close to the forest margins. The preferential occurrence of first infection events in areas with fragmented forests suggests that fragmentation enhances the contacts between humans and infectious disease vectors with no major loss of some putative host species’ habitat.

While this work has shown the existence of significant relationships between forest fragmentation and areas of ebolavirus spillover to human populations, we can only speculate on the underlying mechanisms (exposure to wildlife, bushmeat consumption, habitat destruction, biodiversity loss). The fact that spillover tends to occur in hotspots of forest fragmentation rather than in clearcut areas suggests that chances of human interactions with host wildlife are higher in areas where human encroachment leaves forest fragments that provide habitat for reservoir species.

Does the notion of increased contacts with wildlife imply that human settlements are moving closer to the forest margins? Our analysis based on maps of populated areas (i.e., settlements) available for Central Africa (see Methods section) shows that the average distance between human settlements and both forest margins, which include edge, perforated, patch sites, and smaller forest cores (<200 ha, see Methods) and larger forest cores (>200 ha), has increased between 2000 and 2014 (Table S5), indicating that the ongoing increase in forest loss and fragmentation is associated with a shift of the forest margins away from human settlements rather than the encroachment of villages and inhabited areas into the forest.

The impact of forest loss on ecosystems and the services they provide is often evaluated in terms of habitat destruction, losses of biodiversity, carbon stock and emissions, land degradation, or altered climate and hydrologic conditions^16^,^47^,^48^. This study, however, highlights that deforestation and forest fragmentation potentially have another important class of externalities associated with global health and zoonotic disease outbreaks^15^,^16^,^49^. These externalities should be accounted for while evaluating the costs, risks, and benefits of human encroachment in forested areas. It is also important to understand the interactions existing among the unwanted effects of forest loss and fragmentation; for instance, biodiversity losses may enhance the likelihood of zoonotic infections through increasing the abundance of some species and thus the infection prevalence of specific pathogens through increased intra-specific host contacts and infection transmission^50^. By reshaping forest boundaries, altering habitat and reducing biodiversity^51^,^52^, the growing global pressure on land and its products is increasing the risk of zoonotic infections with important impacts on human health worldwide^53^.

## Methods

### Data

Human *Ebolavirus* cases were obtained from weekly reports by the World Health Organization^54^ as well as a record of locations of first infection reported in Refs 8,31). The centers of early infection (Table 1) considered in this study refer to the period subsequent to 2004, which allows us to determine forest cover, forest fragmentation and their changes in years prior to the outbreaks using forest cover data from Hansen *et al*.^30^ that are available for 2000–2014. Such data are provided as a tree cover map for the year 2000, and annual changes (i.e., both forest loss and gain) until 2014 at the resolution of 30 m. These maps are based on multispectral satellite data (Landsat 7 with ETM + sensors)^30^. In this data set tree cover refers to vegetation taller than 5 m. Information on land use (1 km resolution) was based on new cropland maps for the year 2005 developed by the International Institute for Applied System Analysis and the International Food Policy Research Institute^55^. These maps combine a variety of satellite data sources validated with high-resolution crowdsourcing data^55^. Human settlement data were available from the United Nations Office for Coordination of Human Affairs (OCHA). In this study the “Populated Places (Settlements)” dataset available in the “Common Operational Datasets” was used (https://data.humdata.org/) for Cameroon, Central African Republic, Gabon, Republic of Congo, Democratic Republic of Congo, and Uganda. Population data were available at 1 km resolution from the WorldPop datasets (http://www.worldpop.org.uk/)^56^.

### Data Analyses

The use of the forest loss/gain data^30^ in other tropical forests (e.g., Indonesia) has highlighted the need for validation with ground based observations^57^. In fact, because tree plantations can be confounded with forests, plantation growth or harvest could be mistakenly interpreted as forest loss or gain, respectively. However, by including plantations within the forest classification we simply underestimate the anthropogenic disturbance on core forest areas. We evaluate the ability of these data to provide a reliable representation of forest cover change by using a new cropland cover map (1 km resolution)^55^. For the purposes of this analysis, it was important to evaluate the consistency between areas reported as forest in the forest cover data^30^ and non-crop areas in the cropland maps^55^. To that end, the 30 m forest cover data^30^ were upscaled to 1 km resolution and each 1 km pixel was classified as forest when its tree cover exceeded 80%. The consistency between the two data sets was evaluated for the case of West Africa (Fig. 1b) and the results show that in 99% of the cases (in the entire West Africa subregion) forested areas coincided with areas with no cropland.

Fragmentation analyses were performed using the approach by Vogt *et al*.^58^, as follows: the landscape is represented as a square lattice with 30 m × 30 m pixels. Each pixel is initially classified either as wooded (i.e., with forest trees taller than 5 m) or non-wooded^58^. Wooded pixels that are not adjacent to non-wooded pixels constitute the “forest core”. To evaluate changes in forest fragmentation we count the number of pixels belonging to forest cores with areas <100 ha, >200 ha, and of intermediate size. Wooded pixels that are not adjacent to core pixels form “patches” scattered in a non-wooded background. Similarly, we can define a non-forest core, made of all the non-wooded pixels that are not adjacent to wooded pixels. Wooded pixels that are neither core nor patch pixels belong to a “forest margin”. Margins can be either “edges” or “perforated” areas, depending on whether they are at the forest boundary with non-forest cores or with smaller non-wooded areas, respectively. A threshold of 100 m was used to distinguish perforated from edge areas. We characterize forest fragmentation in a certain area using a composite fragmentation index (CFI), defined as the ratio between the number of pixels classified as “edges”, “perforated”, “patches”, or smaller core areas (i.e., with area <100 ha or between 100 ha and 200 ha), divided by the total number of pixels in that area. CFI varies between 0 and 1; CFI = 1 in areas with extremely fragmented forest cover, while CFI = 0 in areas with no fragmented forest cores or no forest cover at all.

To evaluate how the forest cover and fragmentation existing in the surroundings of centers of first infection compare to the rest of the region, we first investigate changes in forest cover and CFI as a function of the distance from each infection center. To that end, we consider a set of concentric circles (with 25 km, 50 km, 100 km, 200 km, and 300 km radii) centered on each first infection location (Table 1 and Fig. 1). For each circle we determine forest cover and CFI on the years of the infection outbreak (Table 1), as well as the forest loss and increase in fragmentation between 2000 and the infection year. We then compare the values of these indicators of forest structure in the surroundings of the infection centers, with those observed across the region. To that end we randomly sample the forest cover and fragmentation fields by considering 2287 circles (with 25 km, 50 km, 100 km, 200 km, and 300 km radii) randomly placed across the region (196 of which in West Africa, See Figure S2). Using a Mann-Whitney U-test we evaluated whether in the surroundings of the first infection centers forest cover, forest fragmentation, and their changes were on average different from the 2287 randomly placed circles. To eliminate biases associated with the higher population density in the proximity to the centers of first infection, the same test was repeated using only a subset (~1000 random circles) of the 2287 randomly distributed circles with population density comprised within the interquartile range (IQR) of the population surrounding the eleven centers of first infection (or IQR areas; see Results and Discussion Section). In each comparison we verified that the population density in the circles around the first infections points was not significantly different from that in the IQR areas.

It could be argued, however, that an area with a high (or low) value of a particular forest attribute is not necessarily a statistically significant hotspot (of deforestation or fragmentation): to be a hotspot, it needs a high (or low) value of that attribute but it also has to be surrounded by other areas with high (or low) values of the same attribute; otherwise, it would be just an outlier. To that end, circles centered on the first infection locations were used to perform a hotspot analysis applying the Getis-Ord algorithm (Gi* statistics^59^) over a random subselection (2287 circles) of the whole sample. This method is based on stricter criteria than the statistical tests presented in Table 2, in that it accounts for patterns of forest fragmentation in the neighborhood of the first infection centers (i.e., at greater distances than 25 km; see Methods). The Gi* statistic tells whether areas with high or low attribute values tend to cluster and form a hotspot. The result of the Gi* analysis is a statistically significant positive or negative indicator of high value or low value hotspots, respectively. In this work the Gi* analysis uses 25 km radius circles randomly placed within a 250 km distance.

## Additional Information

**How to cite this article:** Rulli, M. C. *et al*. The nexus between forest fragmentation in Africa and Ebola virus disease outbreaks. *Sci. Rep.* **7**, 41613; doi: 10.1038/srep41613 (2017).

**Publisher's note:** Springer Nature remains neutral with regard to jurisdictional claims in published maps and institutional affiliations.

## Supplementary Material

Supplementary Information

## Figures and Tables

**Table 1:** Centers of first infection of Ebola virus disease in humans (data from refs 8,31).

Location	Date Range	Country
1. Yambio	Apr − Jun 2004	South Sudan
2. Odzala	Apr − May 2005	Rep. of Congo
3. Mombo Mounene	May − Nov 2007	Democr. Rep. of Congo
4. Kabango	Aug − Dec 2007	Uganda
5. Luebo	Nov 2008 − Feb 2009	Democr. Rep. of Congo
6. Nakisamata	May 2011	Uganda
7. Isiro	Jul − Nov 2012	Democr. Rep. of Congo
8. Nyanswiga	Jul − Oct 2012	Uganda
9. Luwero District	Nov 2012 − Jan 2013	Uganda
10. Meliandou	Dec - 2013	Guinea
11. Inkanamongo	Aug - 2014	Democr. Rep. of Congo

**Table 2:** Average forest cover, forest loss, fragmentation (CFI), and change in fragmentation in the surroundings of centers of first infection and across the region.

	Average Value (<25 km from infection center)		Regional Average	p-value
**Forest Cover**	0.87	*Entire region*	**0.55**	**0.0052**
		*IQR*	**0.45**	**0.0001**
**Forest Loss**	2.92%	*Entire region*	5.48%	0.9566
		*IQR*	7.66%	0.0501
**Fragmentation**	0.32	*Entire region*	**0.17**	**0.0062**
		*IQR*	**0.20**	**0.0318**
**Change in Fragmentation**	338%	*Entire region*	**296%**	**0.0258**
		*IQR*	**145%**	**0.0033**
**Population density per km**^**2**^	122	*Entire region*	**42**	**0.0117**
		*IQR*	51	0.5766

Regional averages are calculated for the year 2014 in 2287 circular areas (25 km radius) randomly scattered across the entire region, and in the subset of the same 2287 areas that have population density comprised within the interquartile range (IQR) of the population in the surroundings (25 km radius) of the centers of first infection. The p-values are obtained by testing (Whitney-Mann test) that the average values in the surroundings of first infection areas are significantly different from regional averages (highlighted in bold).
