# Supplementary material for: The nexus between forest fragmentation in Africa and Ebola virus disease outbreaks
Source: Sci Rep. 2017 Feb 14;7:41613. doi: 10.1038/srep41613 (PMC5307336; doi:10.1038/srep41613)
Supplement: Supplementary Information [file srep41613-s1.pdf]

The nexus between forest fragmentation in Africa and Ebola virus disease outbreaks

Maria Cristina Rulli[1], Monia Santini[2], David T S Hayman[3], Paolo D'Odorico[4,5]

[1]Department of Civil and Environmental Engineering, Politecnico di Milano, Milan, Italy.

[2]Division on Impacts on Agriculture, Forests and Ecosystem Services, Fondazione Centro Euro-Mediterraneo sui Cambiamenti Climatici Foundation, Viterbo, Italy.

[3]Molecular Epidemiology and Public Health Laboratory (*m*EpiLab), Hopkirk Research Institute, Massey University, Private Bag 11 222, Palmerston North 4442, New Zealand

[4]Department of Environmental Sciences, University of Virginia, Charlottesville, VA, USA.

[5]National Social Environmental Synthesis Center, University of Maryland, Annapolis, MD, USA.

[*]Correspondence to cristina.rulli@polimi.it

Supplementary materials

Figures (Supplementary materials)

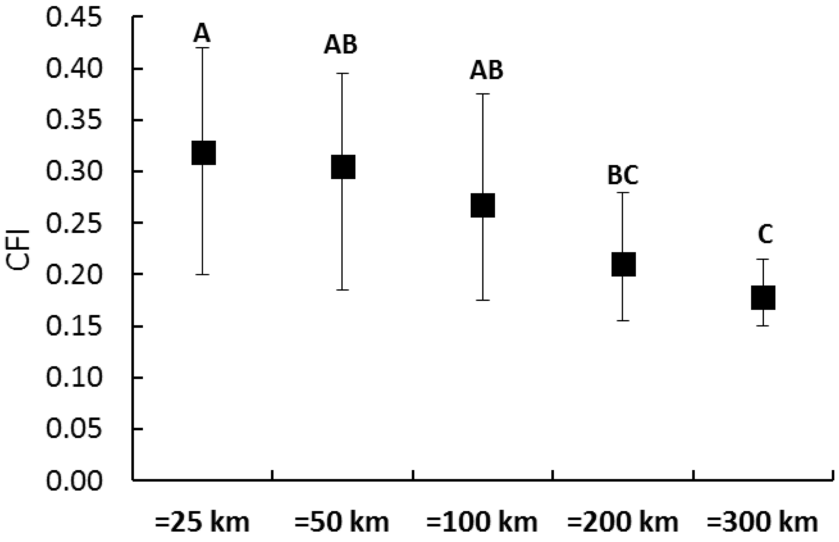

Fig. S1. Average forest fragmentation as a function of the distance. Average forest fragmentation as a function of the distance from the eleven first infection centers (Table S1). Error bars indicate the 25% and 75% percentiles. Different capital letters indicate statistically different means (α=0.05).

Fig. S2. Maps of hot spots of fragmentation Maps of hot spots of fragmentation for a) Central Africa (composite between 2000 and 2014) and b) West Africa (2014) with superimposed outbreaks. Background as in Figure 1. Maps generated by the authors using ARCGIS 10.2-Version 10.2.0.338, licensed to Politecnico di Milano. The license term can be found on the following link: http://www.esri.com/legal/software-license

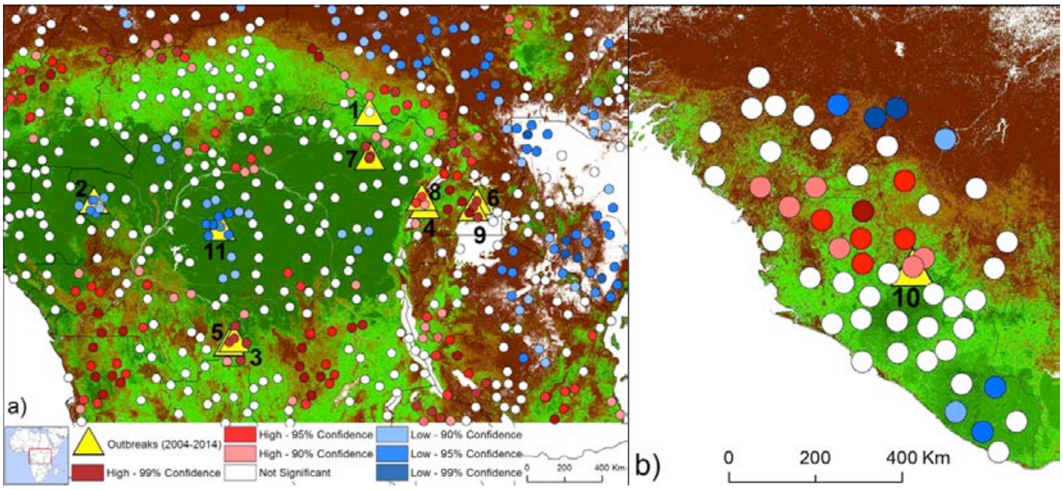

Tables (Supplementary materials)

Table S1. Average forest cover as a function of the distance from centers of first infection. Forest cover is calculated using circles of radius r centered on the infection location, using for each location forest data from the corresponding infection year. The p-values reported in boldface indicate that within a 25 or 50 km distance from the first infection areas the average forest cover is statistically different ($\alpha=0.05$) from the regional mean of [0.55÷0.61] . The regional mean was calculated for the year 2014 by sampling forest cover in 2287 circles with 25 km, 50 km, 100 km, 200 km, and 300 km radii randomly scattered across the region. The p-values (IQR) demonstrate that the first infection areas have a forest cover significantly greater (for distances, r, up to 300 km) than the average forest cover of [0.43÷0.50] in randomly selected areas with population density within the interquartile range (IQR) of the population in first infection areas.

**N.**	**Infection Center**	**Forest cover within a distance, r, from the center of Infection**				
		**r=25 km**	**r=50 km**	**r=100 km**	**r=200 km**	**r=300 km**
1	Yambio	0.99	0.99	0.98	0.92	0.82
2	Odzala	0.99	0.99	0.93	0.87	0.86
3	Mombo Mounene	0.91	0.83	0.77	0.78	0.77
4	Kabango	0.78	0.76	0.71	0.70	0.67
5	Luebo	0.88	0.81	0.76	0.77	0.76
6	Nakisamata	0.85	0.73	0.47	0.31	0.26
7	Isiro	0.91	0.93	0.95	0.97	0.96
8	Nyanswiga	0.81	0.75	0.68	0.65	0.62
9	Luwero District	0.83	0.75	0.49	0.32	0.27
10	Meliandou	0.58	0.75	0.81	0.75	0.62
11	Inkanamongo	0.99	0.99	0.99	0.99	0.97
	Average	0.87	0.84	0.78	0.73	0.69
	p-value	**0.0052**	**0.0301**	0.1532	0.3548	0.2670
	p-value (IQR)	**0.0001**	**0.0001**	**0.0247**	**0.0041**	**0.0066**

Table S2. Average forest loss (%) as a function of the distance from centers of first infection. Forest loss is calculated as the difference in forest cover between 2000 and infection year, using circles of radius r centered on the infection location. The p-values indicate that the average forest loss in first infection areas is not statistically different (α=0.05) from the regional mean forest loss between 2000 and 2014 calculated by sampling forest cover in 2287 circles with 25 km, 50 km, 100 km, 200 km, and 300 km radii randomly scattered across the region (mean forest loss = [5.48%÷3.13%]). Similar results (p-value (IQR)) are obtained by sampling a sub-set of random circles (mean forest loss = [7.66%÷4.14%]) that have population density within the interquartile range (IQR) of the population density in the areas of first infection.

		Forest loss (%) between 2000 and infection date within a distance, r				
**N.**	**Infection Center**	**R=25 km**	**R=50 km**	**R=100 km**	**R=200 km**	**R=300 km**
1	Yambio	0.27%	0.15%	0.16%	0.25%	0.44%
2	Odzala	0.44%	0.30%	0.29%	0.25%	0.26%
3	Mombo Mounene	4.43%	4.50%	4.31%	3.43%	2.56%
4	Kabango	1.97%	1.95%	3.32%	2.70%	2.03%
5	Luebo	8.08%	7.03%	5.84%	4.13%	3.00%
6	Nakisamata	1.79%	3.17%	4.43%	5.09%	4.77%
7	Isiro	7.81%	6.34%	4.36%	2.40%	2.07%
8	Nyanswiga	1.86%	2.78%	4.65%	4.92%	3.46%
9	Luwero District	2.34%	3.52%	4.42%	5.26%	4.51%
10	Meliandou	1.74%	2.35%	3.21%	3.16%	3.85%
11	Inkanamongo	1.38%	1.68%	1.60%	1.96%	2.27%
	Average	2.92%	3.07%	3.33%	3.05%	2.66%
	p-value	0.9566	0.7704	0.4666	0.8250	0.6401
	p-value (IQR)	0.0501	0.0565	0.6255	0.1959	0.0507

Table S3. Average fragmentation as a function of the distance from centers of first infection. Fragmentation is expressed by the CFI index (see Methods) and calculated using circles of radius r centered on the first infection location. Average values reported in boldface indicate that they are statistically different (p-value < 0.05) from the mean regional CFI value of [0.17÷0.15], calculated by randomly sampling CFI in 2287 circles with 25 km, 50 km, 100 km, 200 km, and 300 km radii randomly scattered across the region. The average CFI in the surroundings of areas (r ≤ 100km) of first infection is still significantly different (p-value (IQR) < 0.05) from the regional mean CFI = [0.20÷0.16] calculated considering the sub-sample of random circles having population density within the interquartile range (IQR) of that of the first infection areas. The statistical analysis of fragmentation hotspots for the first infection locations is based on the Getis-Ord method (59) and the results are reported in the last column with significance levels of 0.90 (*), 0.95 (**), and 0.99 (***).

**N.**	**Infection Center**	**Average CFI within a distance, *r*, from infection center**					**Fragmentation Hotspot?**
		***r*=25 km**	***r*=50 km**	***r*=100 km**	***r*=200 km**	***r*=300 km**	
1	Yambio	0.07	0.06	0.09	0.16	0.17	Yes
2	Odzala	0.03	0.03	0.06	0.07	0.07	No
3	Mombo Mounene	0.31	0.37	0.32	0.29	0.25	Yes***
4	Kabango	0.31	0.29	0.32	0.27	0.20	Yes**
5	Luebo	0.37	0.41	0.37	0.30	0.25	Yes***
6	Nakisamata	0.58	0.53	0.36	0.22	0.18	Yes***
7	Isiro	0.46	0.38	0.27	0.15	0.13	Yes**
8	Nyanswiga	0.38	0.38	0.38	0.31	0.23	Yes*
9	Luwero District	0.62	0.53	0.38	0.23	0.19	Yes***
10	Meliandou	0.31	0.28	0.26	0.24	0.20	Yes*
11	Inkanamongo	0.09	0.09	0.08	0.08	0.09	No
	Mean	***0.32***	***0.30***	***0.26***	0.21	0.18	
	p-value	**0.0062**	**0.0047**	**0.0072**	0.0562	0.5252	
	p-value (IQR)	**0.0318**	**0.0451**	**0.0347**	0.1565	0.9423	

Table S4. Average change in fragmentation (%) as a function of the distance from centers of first infection. Change in fragmentation is calculated using circles of radius r centered on the infection location. Average changes in CFI in the first infection points (within a distance r≤25km) is significantly different from the regional mean of 296% calculated between 2000 and 2014 in 2287 circles of 25 km radius randomly scattered across the region.

The p-values (IQR) reported in boldface indicate that changes in CFI are significantly (p-value (IQR) < 0.05) higher (for distances, r, up to 100 km) than the mean regional value ([142÷70]) calculated in circular areas of radius r (25 km, 50 km, 100 km, 200 km, and 300 km) randomly scattered across the region with the condition that in every circle the population density is comprised within the interquartile range (IQR) of the population density in the surroundings (distance ≤r) of the centers of first infection.

**N.**	**Change in CFI (%) between 2000 and infection date within a distance, r**					
	**Infection Center**	**r=25 km**	**r=50 km**	**r=100 km**	**r=200 km**	**r=300 km**
1	Yambio	114	60	27	13	14
2	Odzala	579	151	41	26	38
3	Mombo Mounene	174	122	110	98	73
4	Kabango	45	48	62	48	45
5	Luebo	222	145	124	102	74
6	Nakisamata	28	33	25	27	21
7	Isiro	2111	1637	875	397	265
8	Nyanswiga	43	41	56	62	52
9	Luwero District	30	37	25	28	21
10	Meliandou	7	22	38	34	35
11	Inkanamongo	370	299	277	289	190
	Average	338	236	151	102	75
	p-value	**0.0258**	0.0911	0.1414	0.4825	0.3907
	p-value (IQR)	**0.0033**	**0.0258**	**0.0446**	**0.04537**	0.9446

Table S5. Change (%) in the mean distance between fragmentation sites. Change (%) in the mean distance between fragmentation sites (i.e., patch, edge, perforated, and smaller forest cores, see Figure S2 and Methods) to the nearest settlement using circles of different search radius (25, 50, 100 km) around the outbreak centers. Increasing and decreasing distance trends are found with 90% (*), and 99% (**) confidence.

	**Change in distance (%)**		
**Circle Radius**	**25 km**	**50 km**	**100 km**
Patch	6**	4**	4**
Edge	7**	5**	5**
Perforated	14**	7**	5**
Core < 100 ha	18**	6**	7**
100 ha ≤ Core ≤ 200 ha	26**	13*	-1
Core > 200 ha	15**	13**	15
